# Assessing the Sensitivity of OCT-A Retinal Vasculature Metrics

**DOI:** 10.1167/tvst.12.8.2

**Published:** 2023-08-02

**Authors:** Jacob Szpernal, Mina Gaffney, Rachel E. Linderman, Christopher S. Langlo, Katherine Hemsworth, Ashleigh Walesa, Brian P. Higgins, Richard B. Rosen, Toco Y. P. Chui, Joseph Carroll

**Affiliations:** 1 School of Medicine, Medical College of Wisconsin, Milwaukee, WI, USA; 2 Joint Department of Biomedical Engineering, Marquette University and Medical College of Wisconsin, Milwaukee, WI, USA; 3 Department of Cell Biology, Neurobiology, and Anatomy, Medical College of Wisconsin, Milwaukee, WI, USA; 4 Department of Ophthalmology and Visual Sciences, Medical College of Wisconsin, Milwaukee, WI, USA; 5 Department of Internal Medicine, Ascension St. Joseph Hospital, Milwaukee, WI, USA; 6 New York Eye and Ear Infirmary of Mount Sinai, Icahn School of Medicine at Mount Sinai, New York, NY, USA

**Keywords:** optical coherence tomography angiography (OCT-A), biomarker, vasculature

## Abstract

**Purpose:**

The purpose of this study was to examine the sensitivity of quantitative metrics of the retinal vasculature derived from optical coherence tomography angiography (OCT-A) images.

**Methods:**

Full retinal vascular slab OCT-A images were obtained from 94 healthy participants. Capillary loss, at 1% increments up to 50%, was simulated by randomly removing capillary segments (1000 iterations of randomized loss for each participant at each percent loss). Thirteen quantitative metrics were calculated for each image: foveal avascular zone (FAZ) area, vessel density, vessel complexity index (VCI), vessel perimeter index (VPI), fractal dimension (FD), and parafoveal intercapillary area (PICA) measurements with and without the FAZ (mean PICA, summed PICA, PICA regularity, and PICA standard deviation [PICA SD]). The sensitivity of each metric was calculated as the percent loss at which 80% of the iterations for a participant fell outside of two standard deviations from the sample's normative mean.

**Results:**

The most used OCT-A metrics, FAZ area and vessel density, were not significantly different from normative values until 27.69% and 16.00% capillary loss, respectively. Across the remaining metrics, metric sensitivity ranged from 6.37% (PICA SD without FAZ) to 39.78% (Summed PICA without FAZ).

**Conclusions:**

The sensitivity of vasculature metrics for detecting random capillary loss varies substantially. Further efforts simulating different patterns of capillary loss are needed for comparison. Additionally, mapping the repeatability of metrics over time in a normal population is needed to further define metric sensitivity.

**Translational Relevance:**

Quantitative metrics vary in their ability to detect vascular abnormalities in OCT-A images. Metric choice in screening studies will need to balance expected capillary abnormalities and the quality of the OCT-A images being used.

## Introduction

Optical coherence tomography angiography (OCT-A) is a noninvasive imaging tool that allows visualization of the retinal vasculature in vivo. Retinal vasculature is known to be altered in a variety of ocular,^1^^–^^4^ systemic,^5^^–^^10^ and neurodegenerative diseases.^11^^–^^16^ Although some of these alterations are striking,^10^^,^^17^ other changes can be rather subtle.^11^^,^^18^^,^^19^ These more subtle changes require quantitative or statistical metrics to detect. There is a wide range of metrics used to assess the retinal vasculature, with many focusing on the foveal avascular zone (FAZ), such as the FAZ area. Other metrics focus on the parafoveal vasculature, which includes vessel density across the whole image or within parts of the Early Treatment Diabetic Retinopathy Study (ETDRS) grid, vessel tortuosity, fractal dimensional analysis, vessel complexity index (VCI), and parafoveal intercapillary area (PICA).^10^^,^^12^^,^^15^^,^^20^^–^^22^

With numerous metrics that can quantify the retinal vasculature, it can be difficult to identify the best metric for a specific study. It may be tempting to assess all vasculature metrics within a given population and identify specific metrics that are significantly altered. Whereas this type of exploratory research is necessary at times, such an approach is not feasible for use in a clinical trial or other more structured research studies, as it would be detrimental to its statistical power. Therefore, as OCT-A imaging becomes integrated into clinical trials and routine clinical evaluations, it is important to determine the sensitivity and specificity of the various metrics available. Multiple studies have shown altered retinal vasculature in diseased states with good to excellent sensitivity when compared to a control population.^23^^–^^26^ However, cross-sectional studies do not easily allow for researchers to know when a metric begins to reliably detect abnormalities in the retina as they often include patients at various disease states and by definition do not have baseline data (which is problematic given the large normal variation in the retinal vasculature and FAZ). Understanding which metrics would be best utilized at the initial onset of disease due to higher levels of sensitivity versus which metrics would be best utilized in situations with poor image quality due to their robust nature is critical for further implementation of OCT-A imaging to clinical settings.

There are numerous factors that can affect retinal vascular metrics. For example, factors such as tear film integrity, nystagmus, or the presence of a cataract impact image quality which can affect the reliability and repeatability of vasculature metrics.^27^^–^^29^ In addition, small reductions in blood flow velocity can occur in individuals with retinal disease as well as healthy individuals.^20^^,^^30^^,^^31^ This could be mistaken for nonperfusion or decreased vessel density in a single image, which can confound analyses designed to detect significant differences in retinal vasculature across individuals. Additionally, poor image quality may also be affected by factors with device utilization, such as low signal strength intensity and/or excess motion.^32^ All of the above factors likely do not affect all metrics equally. Therefore, the goal of this study was to simulate random retinal capillary loss to assess the relative sensitivity of different retinal vasculature metrics. The 13 metrics chosen for this study were selected for various reasons. The FAZ area and vessel density were selected due to their prevalent use in the literature and good to excellent sensitivity in altered disease states when compared to a control population.^24^^,^^25^ PICA measurements (mean, sum, SD, and regularity [mean/SD]) were chosen as a result of their abilities to characterize the entire region of interest (ROI). To account for variability inherent among the FAZ in normal populations, PICA-associated measurements were taken both with and without the FAZ.^33^^–^^37^ To evaluate metrics that do not require appropriate scaling of images, we included VCI, vessel perimeter index (VPI), and fractal dimension (FD), which have shown increasing prominence in the literature.^10^^,^^22^^,^^38^^–^^40^

## Methods

### Research Participants

Ninety-four participants (male participants, *n* = 24, 25.53% and female participants, *n* = 70, 74.47%), ranging in age from 11.63 to 66.25 years old (mean ± SD age = 30.93 ± 13.56 years) were included in this study. Self-reported race/ethnicity of the participants was as follows: White/non-Hispanic (*n* = 63, 67.0%), Asian/non-Hispanic (*n* = 14, 14.9%), Black/non-Hispanic (*n* = 8, 8.5%), White/Hispanic (*n* = 3, 3.2%), White/American Indian/Black/non-Hispanic (*n* = 2, 2.1%), and 1 (1.1%) each of American Indian/Hispanic, Native Hawaiian/White/non-Hispanic, American Indian/White/non-Hispanic, Asian/White/non-Hispanic. This study was approved by the Institutional Review Board of the Medical College of Wisconsin (PRO23999) and conducted in accordance with the tenets of the Declaration of Helsinki. Informed consent was obtained from all participants or by the participants’ legal guardian if under the age of 18 once the nature and risks of the study were explained. An ocular health questionnaire was used to collect self-reported ocular history and the Neitz color vision test was performed to test for potential color vision deficits.^41^ Exclusion criteria for the study included participants with self-reported ocular or systemic disease, an abnormal color vision test or children under the age of 5 years. Axial length was acquired in all participants using an IOL Master (Carl Zeiss Meditec, Dublin, CA).

### OCT-A Imaging

The right eye of each participant was imaged using Optovue's Avanti RTvue XR System (Fremont, CA). Two 3 × 3 mm (nominal size) OCT-A volumes, one in the horizontal direction, and one in the vertical direction, were taken and registered to create a single angiogram volume using Optovue's ReVue software (version 2018.4.0.43). A total of five of these registered volumes were acquired for each participant and the full retinal vascular slab (defined as the inner limiting membrane to 9 µm below the outer plexiform layer) was exported as 304 × 304 pixels in tif images. Additionally, the foveal center within each image was automatically determined by the ReVue software and saved.

### OCT-A Image Processing

#### Creating Averaged OCT-A Images

**Figure 1.**
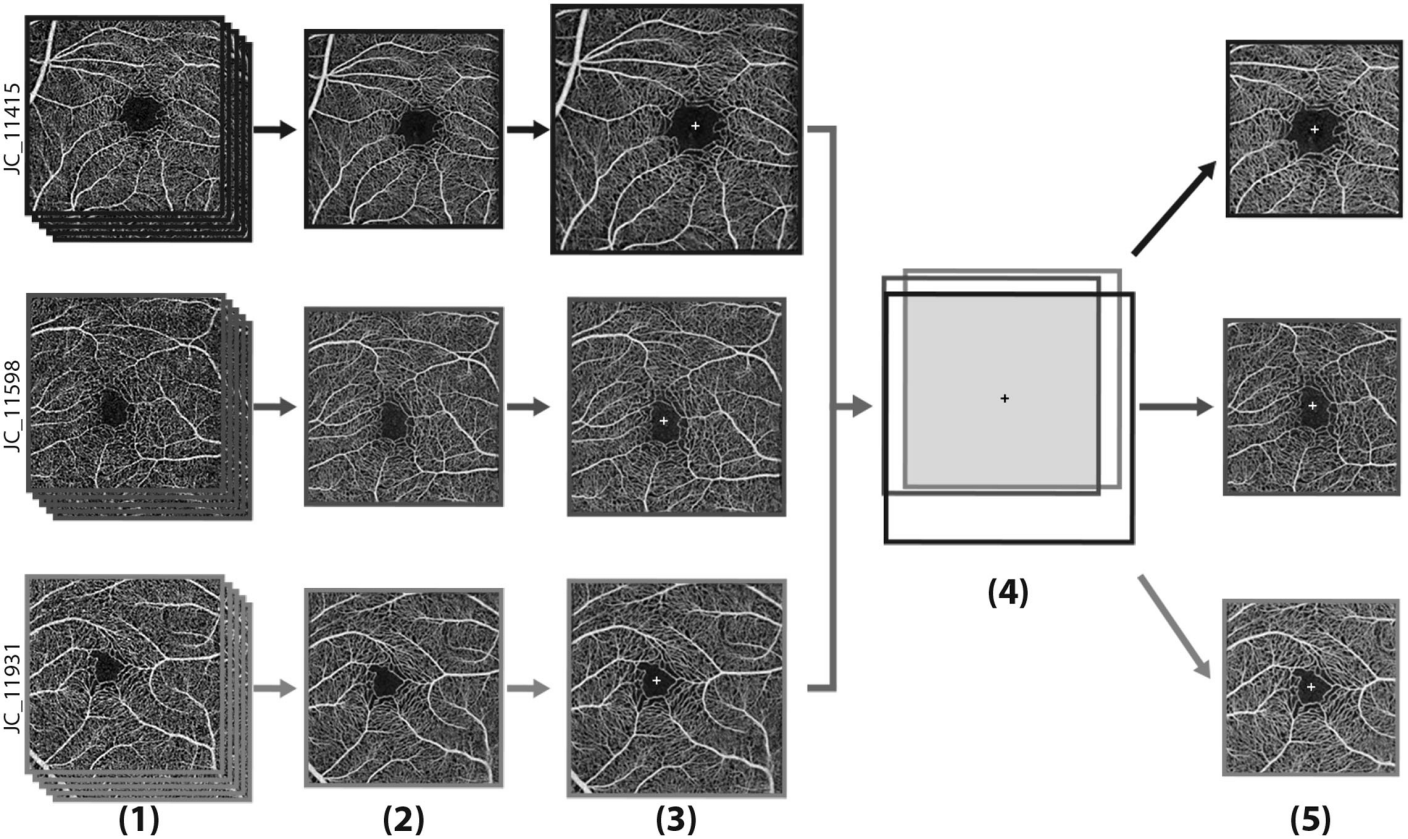
**OCT-A scaling and alignment workflow.** (**1**) Five images were exported from Optovue's Avanti Revue XR system and saved as tif images. (**2**) These five images were registered using bUnwarpJ in Fiji and then averaged together using Z-project in ImageJ and cropped to 304 by 304 pixels. (**3**) All averaged images were then set to the same scale. (**4**) These images were then aligned (*wire diagram*) using the reported foveal centers (*white cross*) from Optovue's ReVue software. Once the images were aligned, the largest common 2380 × 2380 um square area (*shaded region*) was cropped from each image. (**5**) The cropped images were resized to be 304 by 304 pixels. Three participants are shown for illustration purposes: JC_11931 (12-year-old boy), JC_11598 (15-year-old girl), and JC_11415 (25-year-old woman), although the process included all 94 participants.

Figure 1 outlines the initial image processing steps. For each participant, the five angiograms were registered to each other using the best image (determined by author R.E.L.) as the reference frame using bUnwarpJ^42^ in Fiji (National Institute of Health, Bethesda, MD).^43^ Although a single image may be more representative of clinically available images, we wanted to isolate the theoretical metric sensitivity, so the use of averaged images with higher signal to noise helped minimize the contribution of image quality variation. Once all images were registered, they were averaged using Z-project in ImageJ,^44^ and the foveal center was manually marked (by author R.E.L.). The averaged images were then cropped to 304 × 304 pixels and saved as tif images for further analysis. After cropping, the pixel location of the foveal center was recalculated based on image dimensions.

#### Resizing and Rescaling OCT-A Images to a Common Scale

All custom scripts mentioned below can be found in the following repository: https://github.com/AOIP-Lab/Szpernal_etal_OCTA_RetinalVascMetrics. Each averaged image was resized and rescaled to a common scale using a custom MATLAB (MathWorks, Natick, MA) script (OCTA_AlignAndScale.m). This was done by first identifying the smallest axial length among all participants (21.39 mm), then rescaling the averaged OCT-A image for a given participant by the ratio of their axial length divided by the smallest axial length. The pixel location of the foveal center for each image was then recalculated to adjust for the newly resized image by multiplying the *x* and *y* coordinates of the previously calculated foveal center for each participant by their rescaling ratio. The newly scaled images were then aligned to each other using the foveal centers and cropped to the largest common area (2380 × 2380 µm). Each image was then resized to 304 × 304 pixels for a final image resolution of 7.83 µm/pixel for all images.

#### OCT-A Image Preprocessing

**Figure 2.**
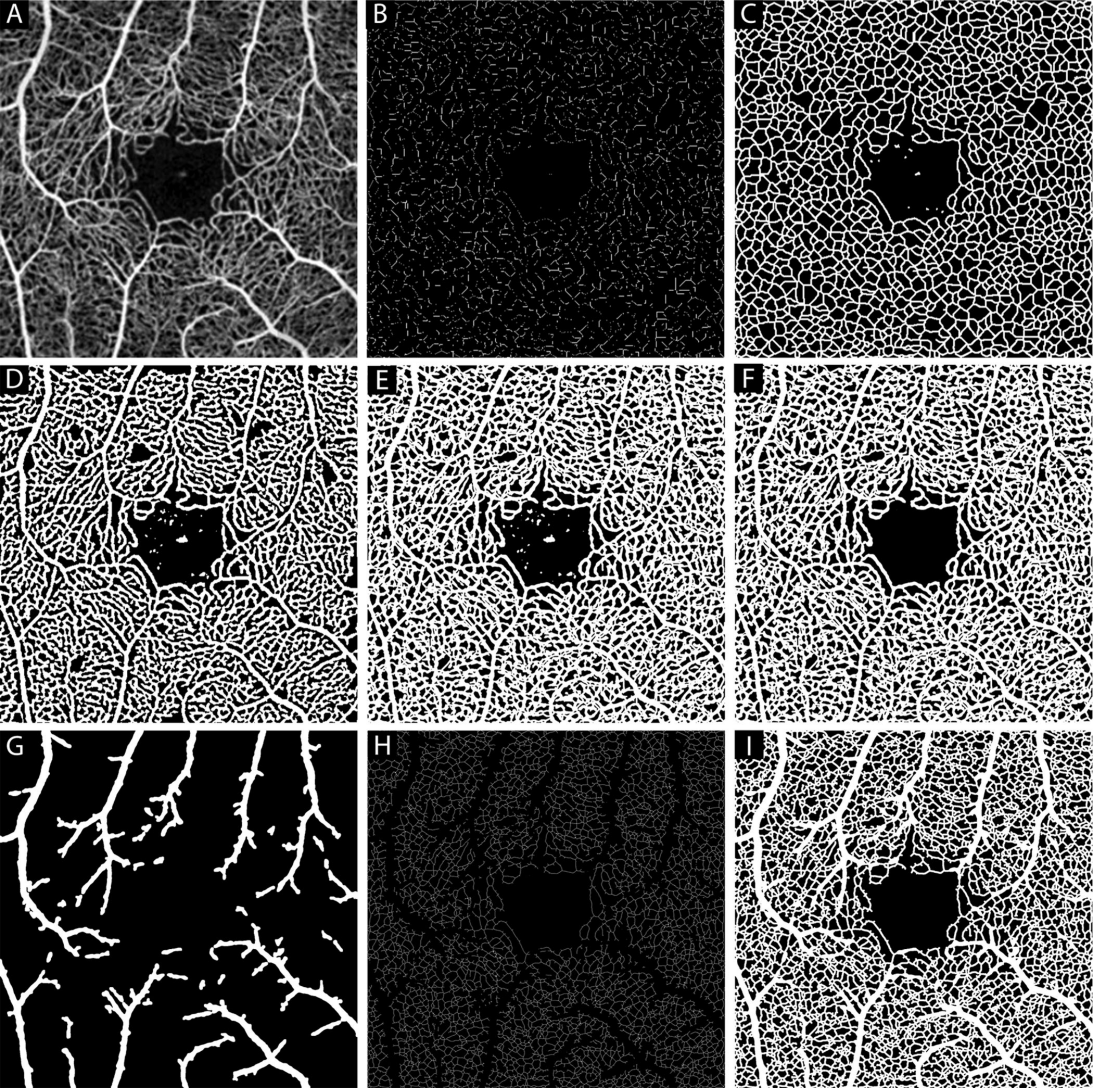
Example of the OCT-A image processing pipeline. The aligned and cropped image from each participant was processed using a custom MATLAB script to clean the OCT-A image and convert into a binary image for metric calculations. Shown in panel (**A**) is the image from one representative subject, AD_10055 (26-year-old man) after being contrast stretched and cleaned by subtracting a background image (which was a variant of the input image with any segments with a radius of less than 15 pixels having been removed). (**B**) The image was then contrast stretched again, resized to 1824 × 1824 pixels, thresholded into a binary image, and then skeletonized. (**C**) The skeleton created in panel **B** was dilated using the function *imdilate* with a structuring element disk with a radius of 5 pixels to segment the small underlying vasculature within the original OCT-A image. (**D**) An enlarged contrast stretched version of panel **A** underwent adaptive thresholding to separate the background and foreground of the image, morphological opening to reduce incorrectly segmented pixels, and was further thresholded. Resulting in an image that was optimized for segmentation of the larger blood vessels within the image. (**E**) A skeleton of panel **C** was then combined with panel **D** to generate an image in which all vasculature within the image was segmented from the background. The image was then manually inspected to remove any noise that remained within the FAZ area, resulting in the cleaned version in panel (**F**). Using an enlarged contrast stretched version of panel **A**, a mask was created of the arterioles/venules, resulting in the image in panel (**G**). The inverse of panel **G** was then used to create a second binary image where the larger arterioles and venules were removed, as shown in panel (**H**). This was then multiplied by panel **F** and skeletonized. The skeleton was then dilated with a structuring element disk with radius of 5 pixels and added to the arterioles/venules mask panel **G** to produce the binary image in panel (**I**).

The newly scaled and cropped images from the above steps were then run through a custom MATLAB script (OCTA_processing.m) to perform a series of preprocessing steps on the images (an example of one participant is shown in Fig. 2). The aligned images were first contrast-stretched to the top and bottom 1% of the intensity values within a given image. The background of the image was then estimated by using morphological opening (*imopen*) to remove all non-black pixel segments with a radius of 15 pixels or less. The background was subtracted from the image to aid in regional background noise reduction, producing the image in Figure 2A. After the background subtraction, the resulting image was then contrast stretched again using the same functions and settings as above. The images were then up-sampled using bicubic interpolation to six times their original size (1824 × 1824 pixels). To remove image artifacts and capillary discontinuities, the newly resized image was dual thresholded using the following equation:

$$\begin{array}{c} I\left(x,y\right)\left\{\begin{array}{c} I<20=0\\ I>35=255 \end{array}\right. \end{array}$$

where, I represents the pixel intensity at a given pixel location (x, y). To smooth the thresholded vasculature a 1-pixel sized averaging filter (*filter2* and *fspecial*) was applied. We refer to this as the “smoothed thresholded image.”

#### OCT-A Segmenting for Small Capillaries

The background and foreground were then isolated from the image by using adaptive thresholding (*adaptivethreshold* function; 15-pixel window size).^45^ The resulting image returns ones for pixels that are deemed to belong to the foreground (mostly vasculature) and zeros for everything else. The perimeter of the foreground pixels was then calculated and subtracted to further differentiate the background and foreground. The results were then inverted, and a median filter was applied for further background noise reduction. Once these thresholding steps were complete, the image was inverted again and skeletonized (*bwmorph*). To reduce the noise within the vasculature of the image all connected objects of 10 pixels or less, spurs, and isolated pixels were removed (*bwareaopen*). To further threshold this skeleton, regions in the skeletonized image that correspond to regions within the original image with values below 30 were set to 0. This thresholded skeleton (shown in Fig. 2B) was dilated using the function *imdilate* with a structuring element disk with a radius of 5 pixels to produce the image in Figure 2C, effectively segmenting the smaller underlying vasculature within the original OCT-A image.

#### OCT-A Segmenting for Larger Blood Vessels

Adaptive thresholding (*Adaptivethreshold*) was also performed on the 6X resized contrast stretched OCT-A image using a window size of 30 pixels to separate the background from the foreground of the image. To reduce incorrectly segmented background and foreground pixels from the previous step morphological opening (*bwareaopen*) was then used to remove any connected objects (white pixels) within the OCT-A image that were less than 3000 pixels. To further threshold this image, it was multiplied by an inverse of a mask of the enlarged OCT-A image that was true everywhere where the image had a pixel intensity value greater than 30 (resulting in the image in Fig. 2D).

#### Manually Removing Noise Within the Foveal Avascular Zone and Saving OCT-A Images and Masks

The optimized skeletons for the smaller vessels (see Fig. 2C) and the thresholded, up-sampled OCT-A image that was optimized for visualizing larger blood vessels (see Fig. 2D) were combined to create a binary image (see Fig. 2E). The binary image was then manually inspected and any noise (appearing as discontinuous groups of pixels) that remained within the FAZ was manually removed by one observer (author R.E.L.) using a custom MATLAB graphical user interface (producing the image in Fig. 2F). Once the noise within the FAZ was removed, a mask was saved with only the larger blood vessels visible. This was done by binarizing (*imbinarize)* the stretched average OCT-A image that had been contrast stretched and upscaled in a previous step. Morphological opening (*bwareaopen)* was then used to remove any connected objects 400 pixels or less. The resulting image was then dilated (*imdilate)* with a structuring element disk radius of 6 pixels (producing the image in Fig. 2G). To generate the binary image where the arterioles/venules were removed, the inverse of the arteriole/venule mask (see the inverse of Fig. 2G) was multiplied (*immultiply)* by the cleaned and thresholded image (see Fig. 2F). The resulting binary image with arterioles/venules removed was then skeletonized (*bwmorph*) to produce the image in Figure 2H. Additionally, the binary image with arterioles/venules present (see Fig. 2F) was skeletonized (*bwmorph*) and saved.

### Simulating Capillary Loss

**Figure 3.**
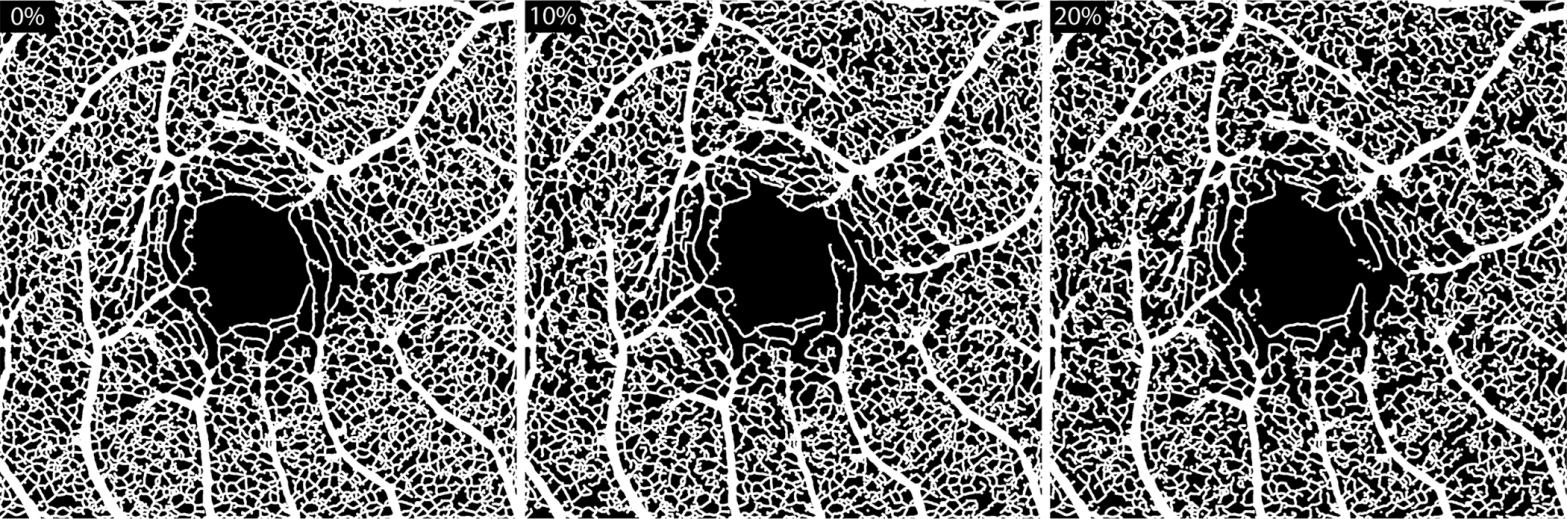
**Depicting random capillary loss.** Shown are three binary images depicting varying degrees of simulated capillary loss (0%, 10%, and 20%) from participant JC_10567 (26-year-old woman).

The masked skeleton (Fig. 2H), original unmasked skeleton (see the skeletonized version of Fig. 2F), and larger blood vessel masks (see Fig. 2G) were then used in another custom script to create randomized capillary segment loss. Randomized intended capillary loss ranged from 1% and 50%, with 1% sampling intervals. For each masked skeleton, the number of capillary segments within the image, the index of every segment, and the coordinates of the segment were computed (*Skel2Graph3D*). Then, 1000 total iterations occurred for each intended capillary loss percentage. The indexed list was randomly permutated causing different segments to be removed at each iteration until the intended percent loss was just exceeded. The segments that were designated to be removed for a given iteration were then subtracted from the original, unmasked skeletonized image. The original unmasked skeleton image, now with random capillary segments removed, was dilated (*imdilate*) using a structuring element disk with a radius of 5. Finally, the mask image of the larger blood vessels (like that in Fig. 2G) was then added to the dilated skeleton to retain the size of the larger blood vessels in the binary image (similar to that seen in Fig. 2I, although this is a binary image with “no-loss”). This binary image was created from each iteration for each subject and saved for further analysis. Depictions of simulated capillary loss are shown in Figure 3 for 0%, 10%, and 20% of intended capillary loss. For depictions of simulated capillary loss across the entire range of intended capillary loss, refer to Supplementary Figure S1.

### Calculating OCT-A Metrics

Each binary image like that depicted in Figure 2I (94 no capillary loss images and the 4,700,000 simulated capillary loss images) was run through another custom script which measured 13 different metrics (OCTA_Metrics.m and OCTA_MetricsCompiler.m). Although these were somewhat arbitrarily chosen, they represent the diverse nature of metrics in use for OCT-A analysis. Vessel density was calculated as the number of white pixels within the binary image divided by the total pixels within the image. The FAZ area and the PICA metrics were automatically delineated and indexed (*regionprops*). For the FAZ area, the nonperfused areas were arranged in descending order and the largest 10 were scanned for the center pixel. The FAZ area was identified as the intercapillary area that included the center pixel (x, y coordinates = 912, 912) of the image.

Four different PICA measurements were calculated once while including and once excluding the FAZ area. For measurements that excluded the FAZ area, metrics were adjusted by excluding the largest nonperfused area associated with the center pixel. Additionally, to account for imperfections obtained from resizing the original (304 × 304 pixels) images to 1824 × 1824 pixels, all PICA areas less than 51 pixels^2^ were excluded from computation. Mean PICA was defined as the mean of all PICA measurements within the image which was found from the area output of the *regionprops* function. Summed PICA with the FAZ area was the total of all the same areas. The standard deviation of all PICA measurements (PICA SD) was the defined as the standard deviation of all PICA measurements and PICA regularity was the mean PICA divided by PICA SD. The PICA measurements which did not include the FAZ area had a prefix of non-FAZ added to them to distinguish the differences of the metrics between these two groups. Using a previously published algorithm,^21^ the VCI and the VPI were also calculated. VCI compares the perimeter of the vessels within an image to the area of the vessels within the image. As described in the original paper,^21^ the VCI is normalized based on a circle that is the same size of the images to remedy the assumption that a VCI of a circle is 1. For our implementation, the VCI was normalized by 1.6169. The VPI is the perimeter of the vessels within the image divided by the area of the image itself.^21^ To find the vessel perimeter, the *bwperim* function with a connectivity of eight was used to delineate the edges of all vessels and subsequently finding the sum of all the pixels in the perimeter image. The sum of all vessel pixels in the image itself was found by adding the total number of white pixels present in the image. Finally, the FD was also completed using the function *boxcount*, another custom MATLAB script.^46^

### Examining the Sensitivity of Retinal Vasculature to Capillary Loss

**Figure 4.**
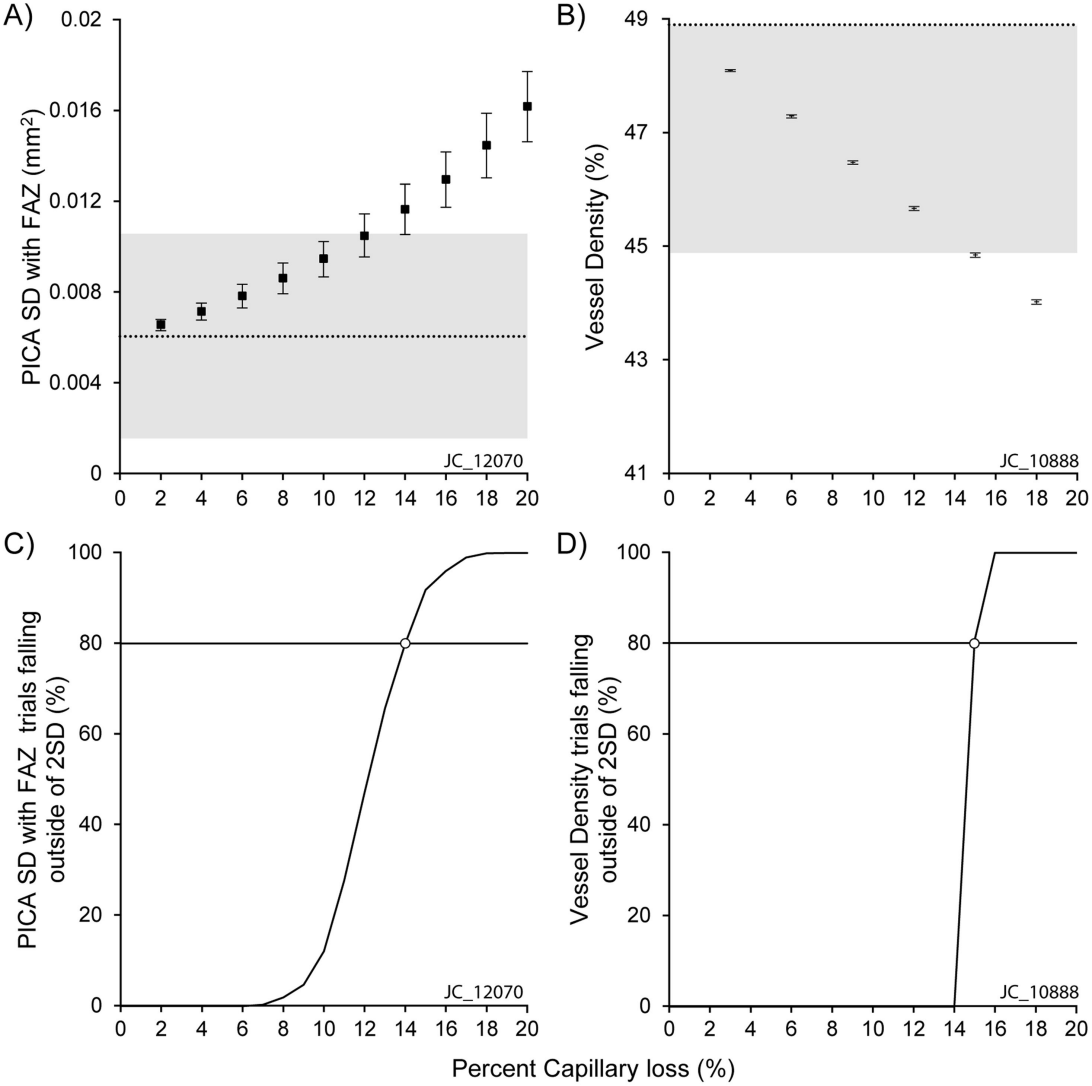
**Depicting sensitivity of PICA SD with FAZ and vessel density in single individuals.** *Top panels* plot the change in absolute metric value as a function of increasing capillary loss for PICA SD with FAZ (**A**) JC_12070 (61-year-old man) and vessel density (**B**) JC_10888 (51-year-old man). The *dotted line* depicts the normative mean of all individuals at 0% capillary loss. The *shaded gray area* depicts ± 2 SD of the normative mean of each metric (note that only the -2 SD is shown for vessel density to improve visibility of the individual data points). Error bars depict the 20th (upper limit) and 80th (lower limit) percentile of the 1000 trials. The metric was deemed sensitive when 800 of the 1000 trials surpassed two SDs. In these examples, 800 trials surpassed two SDs at 14% and 14.99% of intended capillary loss for **A** and **B**, respectively. The *bottom panels* show cumulative frequency histograms for the same individuals – the same respective sensitivities, 14% for JC_12070 (**C**) and 14.99% for JC_10888 (**D**), can be seen where the curve intersects the 80% of the trials line.

For each metric, reference values were established by extracting the metric from the baseline (no capillary loss) images from all 94 participants and computing +/- two standard deviations. For each participant, the baseline value for a given metric was normalized to the average value for that metric across all participants. For each of the 1000 iterations, a z-score, based on the normalized average and standard deviation of the population was calculated for each metric to see if that iteration fell outside two standard deviations of the normal mean for that metric. A metric was deemed sensitive at the intended loss where 80% of all trials fell outside of two standard deviations (Fig. 4). The overall sensitivity for a given metric was calculated as the average sensitivity across all 94 participants for that metric.

## Results

The baseline (no capillary loss) images from the 94 participants were used to create the normative range for this study. The summary statistics for each metric can be found in the Table, with participant data provided in Supplementary Table S1. For each amount of intended capillary loss, the average percent capillary loss was 0.01% greater than intended loss (i.e. 1.01% for 1% intended loss and 50.01% for 50% intended loss). For all data regarding intended versus actual loss, refer to Supplementary Table S2.

**Table.** Normative Metrics Characteristics Across all 94 Participants

Metric	Mean ± SD	Range
FAZ area, mm^2^	0.272 ± 0.102	0.075–0.672
Vessel density, %	48.91 ± 2.01	44.39–54.84
VCI	2949 ± 138	2567–3178
VPI	0.0938 ± 0.0031	0.0867–0.1005
FD	1.862 ± 0.0043	1.851–1.872
Including the FAZ		
Mean PICA, mm^2^	0.0013 ± 0.00016	0.0009–0.0017
Summed PICA, mm^2^	2.895 ± 0.1138	2.559–3.151
PICA regularity	0.242 ± 0.0794	0.106–0.556
PICA SD, mm^2^	0.0060 ± 0.0022	0.00200–0.01526
Excluding the FAZ		
Mean PICA, mm^2^	0.0012 ± 0.0001	0.0009–0.0017
Summed PICA, mm^2^	2.623 ± 0.096	2.398–2.895
PICA regularity	0.7668 ± 0.0548	0.607–0.868
PICA SD, mm^2^	0.0016 ± 0.0002	0.0010–0.0022

Values computed from metric values across all participants with 0% capillary loss.

FAZ, foveal avascular zone; PICA, parafoveal intercapillary area; VCI, vessel complexity index; VPI, vessel perimeter index; FD, fractal dimension; SD, standard deviation.

See Supplementary Table S1 for individual values for all 94 participants.

Average metric values across all 13 OCT-A metrics with increasing capillary loss are provided in Supplementary Table S3. For PICA SD, average PICA, PICA regularity, and summed PICA with the FAZ, respective changes in metric value were +2250%, +931%, −58.1%, and 26.3% when comparing the 50% of intended capillary loss images with the baseline (no capillary loss) image. For PICA SD, average PICA, PICA regularity, and summed PICA without the FAZ, the respective change in metric value was +1767%, +321%, −76.7%, and −45.6% when comparing the 50% of intended capillary loss images with the baseline (no capillary loss) image. Of note, whereas summed PICA with FAZ was noted to increase as the amount of intended capillary loss increased, summed PICA without FAZ was seen to first increase (5.14% at 25% intended loss), then subsequently decrease to −45.6% from the initial metric value at 50% intended capillary loss. This is likely a result of the increasing size of the FAZ area from randomized capillary loss (as the largest nonperfused area with the center pixel is defined as the FAZ) being removed from the final metric calculation.

**Figure 5.**
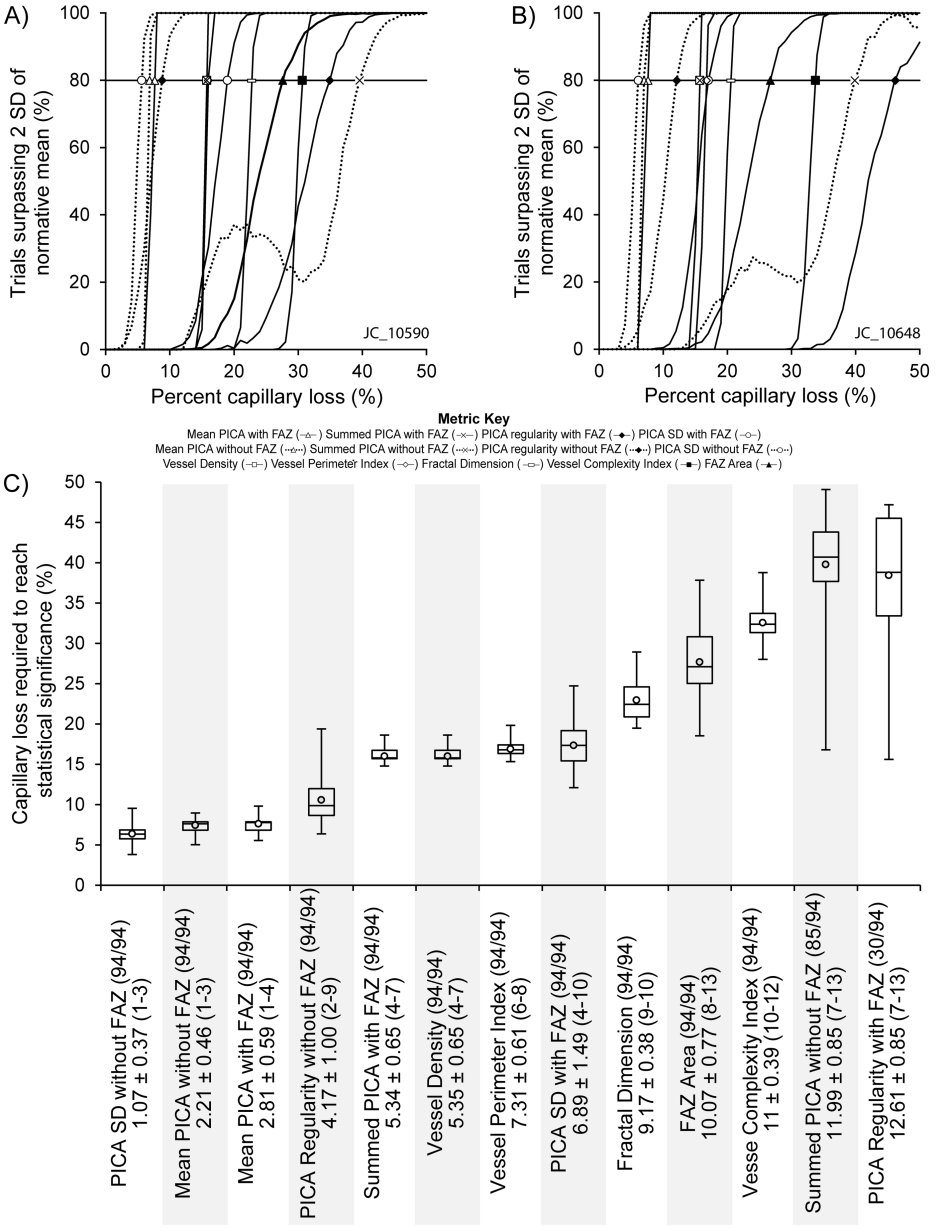
**Relative metric rankings.** Shown are relative metric rankings for two participants: (**A**) JC_10590 (23-year-old woman) and (**B**) JC_10648 (14-year-old man). Each panel depicts the sensitivity at which at least 800 of 1000 trials surpassed two SD of the normative mean for a given metric (*horizontal black line*). Each shape delineates the percent capillary loss at which a metric reached significance. For the participant in panel **A,** the metric sensitivity ranged from 5.62% capillary loss for PICA SD without FAZ to 39.60% capillary loss for summed PICA without FAZ. For the participant in panel **B**, metric sensitivity ranged from 6.13% capillary loss for PICA SD without FAZ to 46.20% capillary loss for PICA regularity with FAZ. Note that some metrics had identical or closely overlapping sensitivity values. For the participant in panel **A** vessel density, summed PICA with FAZ and VPI had sensitivities of 15.77%, 15.77%, and 15.99%, respectively. For the participant in panel **B** vessel density, summed PICA with FAZ, VPI, and PICA SD with FAZ had sensitivities of 15.71%. 15.71%, 16.75%, and 17.05%, respectively. Shown in (**C**) are box and whisker plot characterizing metric sensitivity across all participants. Upper and lower limits of the whisker plot represent the range of capillary loss achieving significance. Exclusive median of first and third quartile depicted as bottom and top of each box, respectively. Mean of each metric depicted as a filled circle inside of the box with median depicted as a horizontal line within the box. The values on the X-axis show the number of participants (#/94) that achieved significance at a capillary loss of 50% or less. The mean rank ± SD and rank range is provided for each metric.

For the remaining metrics, changes in metric value when comparing the 50% of intended capillary loss images with the baseline (no capillary loss) image were 720% (FAZ area), −1.11% (FD), −22.6% (VPI), −17.3% (VCI), and −27.5% (vessel density). Examples of metric sensitivity as a function intended for capillary loss are shown in Figures 5A and 5B, illustrating the variability seen across participants. For JC_10590 (see Fig. 5A), summed PICA without FAZ was seen to be the least sensitive metric (39.60%), whereas for JC_10648 (see Fig. 5B), the PICA regularity with FAZ was the least sensitive metric (46.20%). For JC_10590 and JC_10648, the PICA SD without FAZ (5.62% and 6.13%), the PICA average without FAZ (6.85% and 6.98%), the PICA average with FAZ (7.66% and 7.61%), and the PICA regularity without FAZ (8.79% and 12.09%) were among the most sensitive metrics.

Relative metric rankings across all participants and all metrics are shown in Figure 5C. The PICA SD without FAZ was the most sensitive metric as its average relative rank across all participants was 1.07 with a range of 1 to 3. PICA regularity with FAZ was noted to be the least sensitive, with an average rank of 12.61 and range from 7 to 13. Visual inspection of Figure 5C suggests three tiers of metric rankings with the most sensitive four metrics (PICA SD without FAZ, PICA average without FAZ, PICA average with FAZ, and PICA regularity without FAZ) having average sensitivities of 6.37%, 7.45%, 7.63%, and 10.58%, respectively (“Tier 1”). A second, less sensitive group of metrics (“Tier 2”) include summed PICA with FAZ, vessel density, VPI, and PICA SD with FAZ, with average sensitivities of 16.00%, 16.00%, 16.88%, and 17.34%, respectively. Of note, the identical percent sensitivity between vessel density and summed PICA with FAZ is likely a result of the two metrics measuring the inverse of the other. Last, the least sensitive metrics (“Tier 3”) were FD, FAZ area, VCI, summed PICA without FAZ, and PICA regularity with FAZ, with average sensitivities of 22.96%, 27.69%, 32.55%, 39.78%, and 38.44%, respectively. Of note, only 85 of 94 participants achieved statistical significance in summed PICA without FAZ. This low sensitivity is likely due to the shifting value (increasing then decreasing; see Supplementary Table S3) seen due to the impact of increasing FAZ area with increased capillary loss being subtracted from the metric value, as is seen by the unusual shape of the individual depiction of metric trials in Figure 5A and Figure 5B (dashed line, black “X”). PICA regularity with FAZ also did not achieve significance among all 94 participants with only 30 of 94 achieving statistical significance, suggesting the sensitivity of this metric is greater than 50% capillary loss for 64 of 94 participants. This is likely a result of the large standard deviation seen with the addition of the FAZ in PICA measurements. Additionally, PICA regularity with FAZ displayed a correlation between the size of the FAZ and intended percent capillary loss at which a participant achieved statistical significance (Spearman correlation coefficient *r* = 0.66, *P* < 0.0001, range = 15.63–47.19%).

## Discussion

We assessed the relative sensitivity of 13 commonly used OCT-A metrics that have been previously used to diagnose and/or monitor retinal disease. Each metric's relative sensitivity to randomized capillary loss and the tiered breakdown demonstrated here provide an indication as to which metrics may be most helpful in future studies evaluating capillary loss. For instance, our results show that including the FAZ area in the PICA measurements is a confounding variable. Three of the four most sensitive metrics (Tier 1; see Fig. 5C) exclude the FAZ (PICA SD without FAZ, PICA regularity without FAZ, and PICA average without FAZ). Of note, PICA average with FAZ was also a sensitive metric and this is likely a result of the increasing FAZ rapidly contributing to changes in this value. This is supported by the fact that with 7% of intended capillary loss, PICA average with FAZ increased by 24% with FAZ area increasing by 13.7% (see Supplementary Table S3). More robust (i.e. less sensitive) metrics are otherwise generally seen when the FAZ area is present. For instance, summed PICA with FAZ and PICA SD with FAZ were in the middle tier (Tier 2), while PICA regularity with FAZ and FAZ area itself fell in the least sensitive tier (Tier 3). For PICA regularity with FAZ, the low sensitivity is likely a result of the variability seen with the FAZ area.^34^^,^^47^^,^^48^ In addition, the baseline FAZ area was correlated with the relative capillary loss at which that participant achieved a statistically significant change in PICA regularity with FAZ. Of the 30 participants that achieved statistical significance in PICA regularity with FAZ, the average FAZ area was significantly smaller when compared to the average FAZ found among the 64 participants that did not achieve statistical significance in PICA regularity with FAZ (0.174 mm^2^, 0.318 mm^2^, respectively; Mann-Whitney *U* test *P* < 0.0001).

The FAZ area itself was noted to be in the lowest sensitivity tier likely due to the wide variation in the FAZ area seen across a normal population. This large variability likely resulted in a higher sensitivity threshold for the FAZ area than for other metrics tested.^34^^,^^47^^,^^48^ Metrics in Tier 2 (vessel density and VPI) shared similar sensitivities to each other. This is likely a result of the uniform width of capillaries that were recreated in our simulated capillary loss. Additional metrics in Tier 3 included FD, VCI, and summed PICA without FAZ. For summed PICA without FAZ, the low sensitivity and sinusoidal shape of this curve is likely a result of increasing capillary loss contributing to a more significant FAZ. This results in an increase (from randomized capillary loss contributing to larger non-FAZ PICA areas) and subsequent decrease (because of the expanding FAZ becoming notably enlarged and subsequently deleted from the metric) in trials achieving statistical significance.

These tiers can be used when determining which metrics to use when evaluating OCT-A images for potential abnormalities in retinal vasculature. For instance, when determining whether there are significant abnormalities in the retinal vasculature, one may be more inclined to choose the most sensitive (Tier 1) metrics for early detection. Although Tier 1 metrics may be an attractive metric to use when determining early capillary loss, confounding variables inherently present in OCT-A image acquisition, such as normal and transient perfusion, should be considered.^20^^,^^30^^,^^31^ Other, more robust (i.e. less sensitive) metrics, such as those in the Tier 2, could therefore become more useful in these situations, as detected significant capillary loss will be better protected from confounding variables or lower image quality. Tier 3 metrics and their robust nature may also have clinical utility in instances where OCT-A image quality is expected to vary or decrease significantly.^27^^–^^29^ For instance, the FAZ area delineated on a poor image could offer more clinically useful data regarding disease progression as its robust nature may buffer out imperfections that would inaccurately affect a sensitive metric. However, additional analysis is needed using images with capillary loss and increasing levels of noise before any potential clinical utility can be appreciated. Nevertheless, our data suggest that using multiple metrics from either the same or different tiers may prove beneficial.

There are several limitations to this study. The first regarding scaling of our images. Whereas our approach used OCT-A images that were scaled using a participant's axial length, appropriate scaling of OCT-A images remains rare across most studies. Given this, it is still not known how a metric's sensitivity will change when using unscaled images commonly used in OCT-A studies,^49^ although there are examples in the literature where axial scaling does not impact structure-function correlations.^50^ As such, the scaling aspect is likely not a major limitation. Second, the sample of the data in the present study is relatively small and may not be generalizable to other populations. Specifically, with known variation of retinal vasculature metrics across sex,^51^^,^^52^ race,^53^^,^^54^ and age,^51^^,^^55^^,^^56^ these results may not accurately reflect relative sensitivities in specific demographic cohorts. Third, our method of utilizing randomized capillary loss across the entire image may not accurately reflect capillary abnormalities in vivo. For instance, studies investigating diabetic retinopathy find statistically significant increases in FAZ area when compared to healthy controls and suggest that our findings may underestimate the true sensitivity of metrics involving the FAZ area, although the characteristics of the “healthy control” group can contribute to apparent differences in sensitivity observed between studies.^57^^,^^58^ Certain pathologic processes, including larger vessel loss, abnormalities in vessel tortuosity, and neovascularization in diabetic retinopathy would also affect metric data and would need to be accounted for before clinical interpretation.^59^^,^^60^ Simulating randomized vessel loss also may not coincide with pathologic abnormalities in vivo as studies have shown non-random capillary abnormalities in states of diabetic retinopathy,^61^ sickle cell retinopathy,^62^ and branch retinal vein occlusion.^63^ However, randomized “loss” of capillaries may mimic changes in OCT-A images that result from intermittent flow or variations in image quality, and thus represent a useful tool to probe relative metric sensitivity. Furthermore, various retinal pathologies, such as age-related macular degeneration^64^ and sickle cell retinopathy,^65^ can selectively target different capillary layers within the retina. Because of this, additional studies investigating metric abnormalities should consider replicating specific patterns of loss for specific pathologies. Fourth, although this study highlights potential clinical applications in screening isolated images, it is important to note that absolute and relative metric sensitivities are likely quite different for assessing an OCT-A image when a baseline image for the same patient is available for comparison. Additionally, the analysis performed here applies only to averaged parafoveal images of the full retinal vascular slab. With the emergence of ultra-widefield OCT-A technology,^66^^–^^68^ additional studies investigating the relative sensitivities of metrics would need to be carried out for ultra-widefield images, as the interplay between metrics (i.e. the FAZ area confounding the PICA metrics in this study) would be less pronounced in a larger image. Finally, given the proprietary nature of both image processing and metric derivation by commercially available OCT-A devices, how our analysis extends to images obtained with different devices or to different metric implementations is not known.

Nevertheless, our approach represents a valuable starting point for determining the relativity sensitivities of vascular metrics. Additionally, this study resulted in the production of 4.7 million images with varied states of capillary loss. With continued development in OCT-A associated artificial intelligence (AI)^69^ and machine learning^70^ detecting abnormalities to retinal vasculature, images like these may prove to be useful in training future machine or AI algorithms. Last, this modeling approach could be extended to investigate regional or sectoral capillary loss such as what occurs in a retinal vein occlusion,^71^ retinal melanocytic tumors,^72^ diabetic retinopathy,^73^ sickle cell retinopathy,^62^ or macular telangiectasia.^74^ Further modeling of these and other metrics in isolation and in combination should lead to a better understanding of their respective clinical utility.

## Supplementary Material

Supplement 1

Supplement 2

Supplement 3

Supplement 4
