# Supplementary figures and images for: Assessing the Sensitivity of OCT-A Retinal Vasculature Metrics
Source: Transl Vis Sci Technol. 2023 Aug 2;12(8):2. doi: 10.1167/tvst.12.8.2 (PMC10405864; doi:10.1167/tvst.12.8.2)

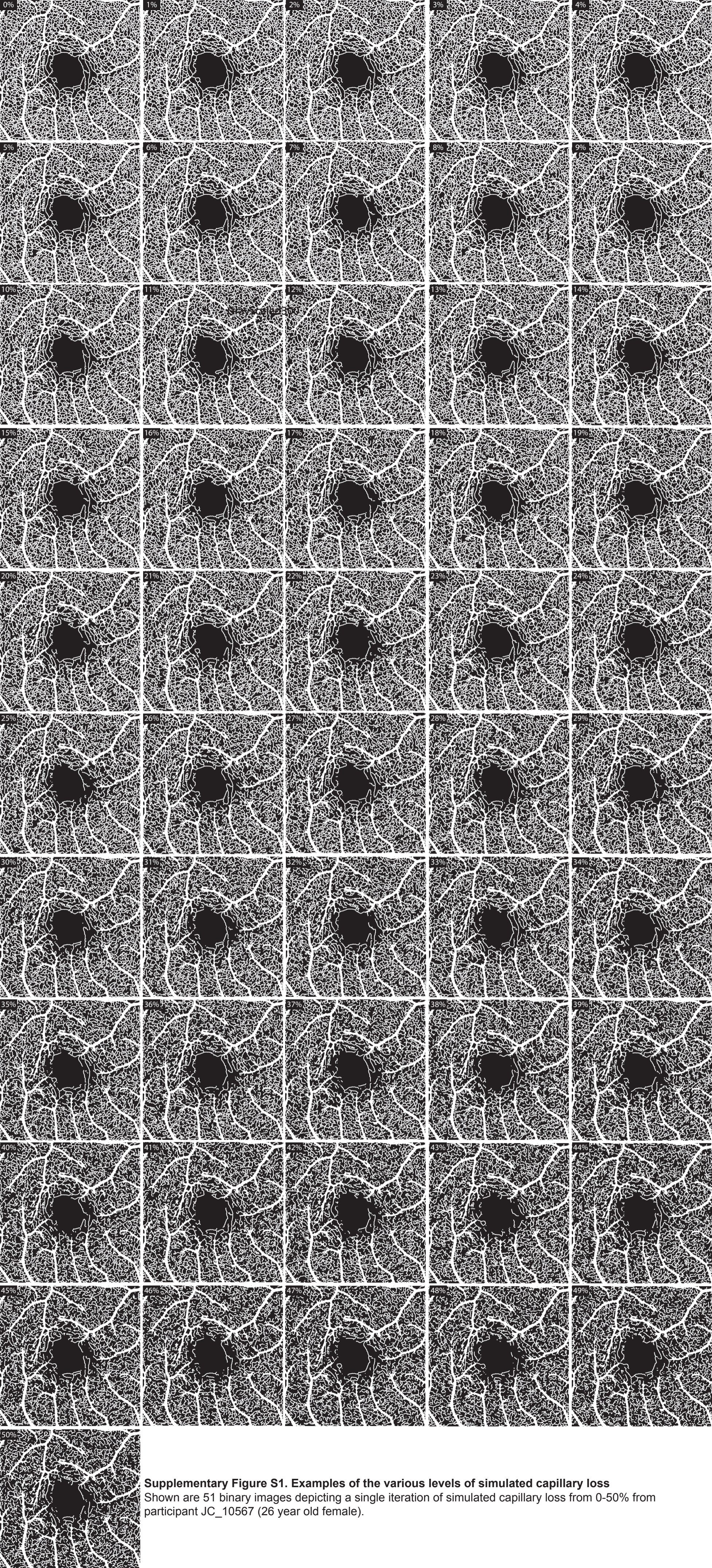

Supplement: Supplement 1 [file tvst-12-8-2_s001.pdf]
